# Treatment of digital dermatitis using salicylic acid in European bison (*Bison bonasus*) reveals promising results

**DOI:** 10.3389/fvets.2022.1012226

**Published:** 2022-12-01

**Authors:** Simone Jucker, Maher Alsaaod, Adrian Steiner, Tatiana Zingre, Sabine Kaessmeyer, Corinne Gurtner, Brian Friker, Sabine Brandt, Tim K. Jensen, Stefan Hoby

**Affiliations:** ^1^Berne Animal Park, Bern, Switzerland; ^2^Clinic for Ruminants, Vetsuisse Faculty, University of Bern, Bern, Switzerland; ^3^Department of Clinical Research and Veterinary Public Health, Vetsuisse Faculty, Institute of Animal Anatomy, University of Bern, Bern, Switzerland; ^4^Department of Infectious Diseases and Pathobiology, Vetsuisse Faculty, Institute of Animal Pathology, University of Bern, Bern, Switzerland; ^5^Department of Clinical Research and Veterinary Public Health, Vetsuisse Faculty, Veterinary Public Health Institute, University of Bern, Bern, Switzerland; ^6^Research Group Oncology, Equine Clinic of Surgery, Departement of Companion Animals and Horses, University of Veterinary Medicine, Vienna, Austria; ^7^Center for Diagnostic, Technical University of Denmark, Kongens Lyngby, Denmark

**Keywords:** *Bison bonasus*, *Treponema spp*., digital dermatitis, salicylic acid, PCR, fluorescent *in situ* hybridization, transmission electron microscopy

## Abstract

Digital dermatitis (DD) associated with the presence of multiple *Treponema* spp. was recently described for the first time in European bison (*Bison bonasus*). DD is characterized by skin inflammation in the distal foot area in various ungulates. The objective of this proof of concept study was to test a treatment protocol adopted from cattle for its applicability in this wildlife species using five animals. Keratolytic salicylic acid paste was administered topically under bandages for seven days to enable removal of the affected skin. All interventions were performed under general anesthesia. To evaluate the treatment efficacy, photographs and biopsies were taken pre- and post-treatment. The biopsies were examined histologically, by PCR for the presence of different bacterial species, by *Treponema*-specific fluorescent *in situ* hybridization (FISH), and by transmission electron microscopy. Based on photographs, complete clinical healing of the 15 feet with macroscopical DD lesions was achieved. Histological examination showed mild to moderate dermatitis in 17/20 feet before, and in 12/20 feet after treatment. 17/20 feet were *Treponema* spp. PCR positive before, and none was positive after treatment. *Dichelobacter nodosus, Fusobacterium necrophorum*, and *Porphyromonas levii* could not be detected in any of the samples. By FISH and electron microscopy, *Treponema* spp. could be visualized in the stratum corneum before, but not after treatment. These results suggest that this treatment method can be applied as standard practice prior to transporting DD affected European bison to prevent the spread of this contagious disease.

## Introduction

The European bison (*Bison bonasus*) or wisent, originally native to large parts of Europe, was extirpated in 1927 (1). Today the population has recovered with a total number of over 9,000 individuals (2). In 2020, the international union for conservation of nature (IUCN) changed the conservation status from vulnerable to near threatened, but with emphasis that the low genetic variability leaves that species still highly susceptible to diseases, which therefore should be monitored carefully (3). With increasing numbers of free-living European bison and only few suitable and protected areas, animal density and thus intraspecies infection pressure increases (4). Additionally, the interaction between European bison and domestic ungulates might be intensified, leading to an emergence of spillover pathogens (4). All free-living herds still need to be managed and release of captive bred animals regularly takes place to establish, increase or restock free-living populations. Therefore, a risk of introducing new pathogens into those sub-populations is a constant threat and must be minimized with appropriate quarantine measures, and treatment or eradication protocols in case of relevant diseases (4, 5).

Digital dermatitis (DD) is an infectious foot disease characterized by proliferative and ulcerative skin inflammation in the distal foot area (6). While DD is endemic in cattle worldwide (7) it also occurs in other ruminants such as sheep (8), goats (9), free-living north American elk [*Cervus elaphus* (10)], and European bison (11). Various bacterial species of the genus *Treponema* are consistently associated with DD lesions, but other bacteria such as *Dichelobacter nodosus* (*D. nodosus*), *Fusobacterium necrophorum* (*F. necorphorum*), and *Porphyromonas levii* (*P. levii*) are also linked to DD lesions (12–15). The exact etiology and pathogenesis of DD are still under discussion. DD is a multifactorial disease, and factors such as genetic predisposition (16), lack of hygiene (17), or poor integrity of the skin (18) are suspected to be involved in the emergence and persistence of the disease. Transmission routes and infectious reservoirs are still not fully understood (19). DD-Treponemes were detected in gastrointestinal host tissue (e.g., rectum, gingiva, rumen), but also in cattle feces and slurry (20–22). Therefore, direct skin-to-skin contact or short-term persistence in slurry are suspected to act as major routes of transmission.

Although DD has been a significant disease for several decades, strategies to eradicate DD from affected farms are unknown (23). In European bison no treatment has been applied yet. Treatment of individually affected cattle consists mainly of topical application of antibiotics or antiseptics with inconsistent success rates (23), but the problem of global antibiotic resistance requires alternative treatment strategies. Salicylic acid (SA) with bactericidal, antiseptic, anti-inflammatory and keratolytic effects has been tested in cattle in the past years with good success rates to treat DD-associated foot lesions (24–26).

The aim of this proof of concept study was to transfer a successful treatment for cattle based on SA bandages to European bison affected by DD in a zoo setting. It was hypothesized that complete clinical and bacteriological healing was achieved by applying SA paste under bandage for 1 week. No negative control group was included as it was not justifiable from an animal ethics point of view since anesthesia is mandatory for each intervention. Treatment efficacy was evaluated by blinded methods clinically, and on a histopathologic and molecular base. In addition, the localization of the spirochetes was investigated ultrastructurally.

## Method

### Ethical statement

This animal experiment was approved by the animal experimentation committee of the canton of Bern (BE10/21, 33227), in accordance with the Swiss animal welfare regulations.

### Animals and housing

The study was conducted from June to December 2021 at Berne Animal Park, where the whole herd of European bison was affected by DD (11). The herd consisted of 12 animals, of which five juveniles of mixed age (15–29 mo.) and sex (m = 3; f = 2) were selected for the study (Table 1). The five breeding animals were excluded due to practical reasons (e.g., limited space in stables, pregnancy). The group was kept in a 5 ha outdoor enclosure.

**Table 1:** Macroscopical and histological scores, PCR and sequencing results, and fluorescent *in situ* hybridization (FISH) scores before (B1) and seven days after treatment (B2) of digital dermatitis lesions in five European bison (*Bison bonasus*).

**Animal No. (^a^sex, age in months)**	**^b^Limb**	**Macroscopical results**		**Histological results**				**Bacteriological results**				^**h**^**FISH results** **(0–3)**	
		^**c**^ **Localization/** **size (0-2)**		^**d**^ **Dermatitis/** **keratinolysis score (0-3)**		^**e**^**Amount of** **spirochetes (0-3)**		^**f**^ **TT PCR**		^**g**^**Amplicon analysis: best hit (%)** **(GenBank accession No.)**		
		**B1**	**B2**	**B1**	**B2**	**B1**	**B2**	**B1**	**B2**	**B1**	**B2**	**B1**	**B2**
1 (f, 29)	LF	1/1	0/0	2/0	1/0	0	0	+	-	*Treponema* phylotype PN-20 (98.00%) (GQ424185)	-	3	0
	RF	0/0	0/0	1/0	1/0	0	0	+	-	n.a.^i^	-	0	0
	LH	0/0	0/0	1/0	1/0	0	0	+	-	*Treponema* phylotype PN-20 (98.99%) (GQ424185)	-	3	0
	RH	1/1	0/0	1/0	1/0	0	0	+	-	*Treponema* phylotype PN-20 (97.85%) (GQ424185)	-	3	0
2 (m, 24)	LF	0/0	0/0	1/0	1/0	0	0	-	-	-	-	0	0
	RF	1/1	0/0	1/0	2/0	0	0	-	-	-	-	0	0
	LH	1/2	0/0	1/0	1/0	0	0	+	-	*Treponema* phylotype PN-20 (98.97%) (GQ424185)	-	2	0
	RH	2/2	0/0	0/0	2/0	0	0	+	-	*Treponema* phylotype PN-20 (98.91%) (GQ424185)	-	2	0
3 (f, 15)	LF	0/0	0/0	1/0	1/0	0	0	+	-	^j^*Treponema* phylotype PT3 (99.29%) (AM942447)	-	0	0
	RF	1/1	0/0	2/0	1/0	0	0	+	-	n.a.	-	0	0
	LH	2/2	0/0	2/2	0/0	1	0	+	-	*Treponema* phylotype PN-20 (99.09%) (GQ424185)	-	3	0
	RH	2/2	0/0	2/1	0/0	0	0	+	-	*Treponema* phylotype PN-20 (100.00%) (GQ424185)	-	3	0
4 (m, 24)	LF	1/1	0/0	0/0	0/0	0	0	-	-	-	-	0	0
	RF	2/2	0/0	0/0	0/0	0	0	+	-	^j^*Treponema* phylotype PN-20 (99.56%) (GQ424185)	-	0	0
	LH	2/2	0/0	1/0	0/0	0	0	+	-	^j^*Treponema* phylotype PN-20 (99.15%) (GQ424185)	-	2	0
	RH	2/2	0/0	1/1	0/0	2	0	+	-	*Treponema* phylotype PN-20 (99.64%) (GQ424185)	-	3	0
5 (m, 16)	LF	1/1	0/0	1/0	0/0	n.a.	0	+	-	*Treponema* phylotype PT3 (99.76%) (AM942447)	-	n.a.	0
	RF	1/1	0/0	1/0	1/0	2	0	+	-	*Treponema* phylotype PT3 (99.81%) (AM942447)	-	2	0
	LH	0/0	1/2	1/0	0/0	0	0	+	-	*Treponema* phylotype PT3 (99.18%) (AM942447)	-	3	0
	RH	1/1	0/0	2/0	1/0	3	0	+	-	*Treponema* phylotype PN-20 (99.43%) (GQ424185)	-	3	0

^a^f, female; m, male.

^b^Limb: LF, left front; RF, right front; HL, hind left; HR, hind right.

^c^The localization of the lesion was categorized into (0) no lesion; (1) focal lesion on the dorsal aspect of the digital skin; (2) extended lesion involving the dorsal aspect of the digital skin and the interdigital cleft. The size of the lesion was classified as (0) no lesion; (1) maximum diameter < 2 cm; (2) maximum diameter ≥ 2 cm.

^d^Modified scoring according to Read and Walker (27) altered by Klitgaard et al. (28). Rating of chronic perivascular and lymphoplasmacytic dermatitis: (0) no changes present; (1) mild; (2) moderate; (3) severe. The degree of keratinolysis was scored as (0) none present; (1) mild; (2) moderate; (3) extensive.

^e^The amount of spirochetes visualized by Warthin Starry stain (WS) on top and in the epidermis was semi-quantitatively classified as (0) non visible; (1) minimal amount; (2) moderate amount; (3) high amount.

^f^*Treponema* PCR: -: negative, +: positive. The quality of DNA was verified in all negative TT PCR sample by a standard β-actin PCR.

^g^Amplicon analysis *via* BLAST nucleotide search: best match of the isolates (identity %) and GenBank accession number for the 16S rRNA gene.

^h^Fluorescent *in situ* hybridization (FISH) for the detection of *Treponema* spp. Scoring of the hybridization signal was performed according to Rasmussen et al. (29) with minor modifications: (0) no hybridization; (1) sparse hybridization; (2) moderate hybridization; (3) strong hybridization.

^i^n.a., not assessable.

^j^Only the 5′ or 3′ strand was compared to BLAST nucleotide search due to low overlap of the two strands.

A footbath (2.7 m length x 0.8 m width x 0.14 m depth) was installed in September 2019 through which the herd has since been passed three times a week. It contained 232 liters of 2% organic acids (EasyStride™, DeLaval, Switzerland) which was renewed according to the manufacturer's instructions when color changed. During the animal experiment, when foot bandages were applied, the footbath was not in use.

### Animal preparation and anesthesia

Each animal was examined by sight 1 day prior and 1 day after each anesthesia for its general and body condition and locomotion. Only animals in normal general condition were included in the study and anesthetized with ketamine and medetomidine according to the protocol described recently (11). Vitamin B_12_ (Catosal 10%, Provet AG, Switzerland; 5.7 mg/kg butafosfan and 0.003 mg/kg cyanocobolamin SC) was routinely administered. Carprofen (Rimadyl Rind, Zoetis, Switzerland; 1.4 mg/kg SC) as well as vitamin E / selenium (Tocoselenit, Gräub, Switzerland; 1mg/kg vitamin E and 0.04 mg/kg sodium selenite SC) were administered when biopsies were taken. Fifty-five to 70 min after induction, anesthesia was partially reversed by administration of atipamezole (Alzane, Gräub, Switzerland; 0.4 mg/kg IM).

### Experimental procedures, treatment protocol, and biopsy collection

**Figure 1:**
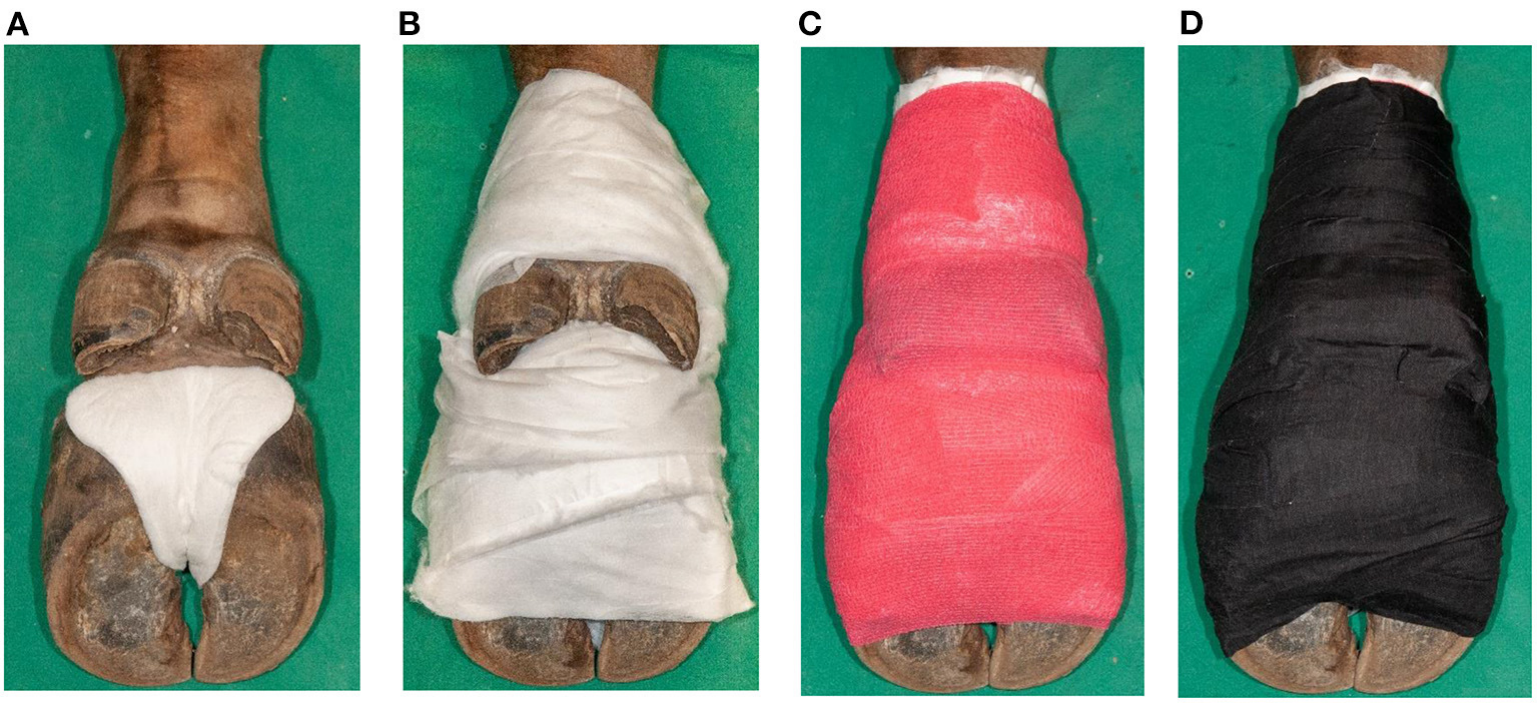
Application of a firm bandage after salicylic acid paste (Novaderma ad us. vet., Paste, Streuli Tiergesundheit AG, Switzerland) treatment of digital dermatitis lesions in European bison (*Bison bonasus*). **(A)** Padding of interdigital cleft with a water-repellent, ergonomic compress (ITIN+HOCH GmbH, Switzerland) **(B)** followed by padding of the skin from the claws until above the dewclaws with omission of the interdigital cleft and dewclaws with absorbent cotton (Kistler cotton with fleece, Covetrus AG, Switzerland). **(C)** Fixation with cohesive tape (cohesive bandages, Covetrus AG, Switzerland) in eight-shaped loops, integrating the interdigital cleft, and in a circular way up until above the dewclaws. **(D)** Double wrapping of the foot including the interdigital cleft with adhesive tape (Tesa 4688, Covetrus AG, Switzerland) to guarantee water-repellent characteristics.

All four feet per animal were included in this proof of concept study and treated with SA paste (Novaderma ad us. vet., Paste, Streuli Tiergesundheit AG, Switzerland; 660 mg SA and 7.7 mg methylsalicylate per 1 gram) under bandages for seven days as described in cattle (24). To monitor the treatment effect, pre- and post-treatment biopsies were taken, requiring three anesthesias per animal. During the first anesthesia, the hair of each foot was clipped before being cleaned with water and dried. Photographs of the dorsal aspect of the interdigital cleft were taken while the claws were splayed manually, followed by skin disinfection with 70% ethanol-soaked gauzes. A sterile punch biopsy (B1, 4 mm diameter, 7 mm depth) was taken perpendicular to the skin surface from the center of the most distinctive lesion. The biopsy was removed under sterile conditions, cut longitudinally into two parts, and stored in an Eppendorf tube at−20°C for PCR and in 10% neutral buffered formalin for histological and FISH examinations. In animal No. 5, each biopsy was cut into three parts for additional storage in 2.5% glutardialdehyde for electron microscopical examinations. Povidone-iodine ointment (Betadine ointment ad us. vet., Covetrus AG, Switzerland) was applied around the biopsy site under a firm water-repellent bandage which was applied by means of a water-repellent, ergonomic compress (ITIN+HOCH GmbH, Switzerland), absorbent cotton (Kistler cotton with fleece, Covetrus AG, Switzerland), cohesive (Cohesive bandages, Covetrus AG, Switzerland), and adhesive tape (Tesa, Covetrus AG, Switzerland) (Figures 1A–D). A healing period of 7 days was chosen between the first biopsy (B1) and the application of SA. The animal was anesthetized after seven days to take off the povidone-iodine bandages. SA was applied (~ 4 mm thickness) onto the DD lesion after cleaning the area with povidone-iodine solution (Betadine solution, Covetrus AG, Switzerland) and water. Milker's fat cream (Eutra Tetina vet., Interlac, Switzerland) was applied adjacent to the SA paste to keep the keratolytic impact of the SA away from the healthy skin before applying another firm water-repellent bandage. All feet were rated as DD affected by clinical inspection and were biopsied and treated with povidone-iodine. After 7 days, the SA bandages were taken off during the third anesthesia, and all loose and necrotic tissue was removed manually from the digital and interdigital skin to expose the newly grown epidermis. Photographs and a second biopsy (B2) were taken as previously described by choosing B2 site next to B1 site. The biopsied area was treated with povidone-iodine ointment and covered by a loose bandage, consisting of a compress that was held in place by cohesive and adhesive tape that fell off within one to 35 days.

### Classification of macroscopical lesions

After the last trial, pre- and post-treatment photographs were blinded and scored by the first (SJ), second (MA) and last author (SH) together. The localization of the lesion was categorized into (0) no lesion; (1) focal lesion on the dorsal aspect of the digital skin; (2) extended lesion involving the dorsal aspect of the digital skin and the interdigital cleft. The size of the lesion was classified as (0) no lesion; (1) maximum diameter < 2 cm; (2) maximum diameter ≥ 2 cm. A lesion was scored negative or cured if the score was 0/0 (localization/size). If there were several lesions per foot, the foot was classified according to the lesion with the highest score.

### Processing and evaluation of histological samples

The fixed samples were routinely trimmed, embedded in paraffin, sectioned at 2 μm and mounted on glass slides. Staining of the sections was performed with hematoxylin and eosin (HE) and a Warthin-Starry silver stain (WS). HE sections were scored for dermatitis and keratinolysis with minor modifications as described earlier (27) and modified according to (28). The occurrence of chronic perivascular and lymphoplasmacytic dermatitis was rated as (0) no changes present; (1) mild; (2) moderate; (3) severe. The degree of keratinolysis in the stratum corneum was scored as (0) none present; (1) mild; (2) moderate; (3) extensive. Additionally, the number of spirochetes visualized by the WS stain on and in the epidermis was classified as (0) non visible; (1) minimal amount; (2) moderate amount; (3) high amount.

### DNA extraction, PCR assays, and sequencing

Samples were blinded before analysis. Using a commercial DNeasy Blood and Tissue kit (Qiagen, Germany), DNA was extracted according to the manufacturer's instructions. Purified DNA extracts were stored at −20°C.

For detection of treponemal DNA, *Treponema* PCR (TT-PCR) assays were performed using *Treponema* primers 5′/3′ (30) and an established PCR protocol (31). The quality of DNA was verified in all negative TT PCR samples by a standard β-actin PCR (32). Electrophoresis was performed with PCR products on 1.5% tris-acetate-EDTA agarose gels and stained with ethidium bromide. As molecular weight marker, a GeneRuler 100 bp DNA ladder (ThermoScientific, Austria) was used. With the QIAex II gel extraction kit (Qiagen, Germany), purification of the amplicon aliquots from the gel was performed following the manufacturer instructions. The amplicons were then shipped for direct bidirectional sequencing (Eurofins, Austria), and the results were compared against GenBank using BLAST alignment (https://blast.ncbi.nlm.nih.gov/Blast.cgi).

Additionally, the samples were tested for *D. nodosus* and *F. necorphorum* DNA according to an established PCR protocol (33). PCR products were visualized on 1.5% tris-acetate-EDTA agarose gels and stained with MIDORI Green Advance. As positive control, skin samples from sheep (*D. nodosus*) and cattle (*F. necrophorum*) were included, sterile water was used as negative control.

The primers used to amplify the 16S rRNA gene fragment of *P. levii* were designed by Primer Blast [National Center for Biotechnology Information (NCBI)]. The downloaded sequence was aligned, and the areas of the consensus sequence closest to the 5′ and 3′ ends were selected. The 5′ primer sequence (named F-Primer 677) was AAGGCAGCTTACAAAAGTGTA and the 3′ primer sequence (named R-Primer 812) TTTCGCTTGAGAGCATACAT. Each PCR contained 10 μl GoTaq^®^ Green Master Mix (Promega AG, Switzerland), 0.1 μl of each primer and 1 μl of PCR template. The reaction mixtures were heated to 95°C for 5 min, cycled 35 times at 95°C for 1 min, 54°C for 1 min, and 72°C for 2 min, followed by incubation at 72°C for 5 min. PCR products were visualized on 2% tris-acetate-EDTA agarose gels and stained with MIDORI Green Advance. Treponema positive skin samples from cattle were used as positive, sterile water as negative control.

### Processing and evaluation of samples for FISH

Sections of 2 μm formalin-fixed, paraffin embedded biopsies were mounted on Epredia ™ SuperFrost Plus™ adhesion slides (Fisher Scientific, Denmark), deparaffinized, and hybridized as described earlier (29), using the oligonucleotide probe for the genus *Treponema* 5′- labeled with Cy3 (No. 202) (28). Hybridization was performed for 16 h at 45 °C and with a final probe concentration of 5 ng/μL.

For examination of the hybridized samples, an Axioimager M1 microscope (Carl Zeiss, Germany), equipped for epifluorescence with a 100-W HBO lamp and filter sets 24 (excitation at 485/578 nm), 38 (excitation at 470 nm), and 43 (excitation at 550 nm) was utilized.

Scoring of the hybridization signal was performed on blinded samples by the second author (MA) according to Rasmussen et al. (29) with minor modifications. The total amount of *Treponema* spp. was scored from 0 to 3: (0) none visible; (1) low number; (2) moderate number and (3) high number visible.

### Processing and evaluation of samples for transmission electron microscopy

Fixation of skin samples was performed in a solution of 2.5% Glutaraldehyde and 0.1 M cacodylate buffer pH 7.4 (Merck KGaA, Germany). Samples where then washed with 0.1 cacodylate buffer and placed 2 h in 1% osmium tetroxide (Polysciences Europe GmbH, Germany) for post-fixation. Dehydration was obtained through an ascending ethanol series. EPON resin was used for embedding and DMP 30 (Polysciences Europe GmbH, Germany) as catalyst. The embedded samples were then left 5 days at 60°C for polymerization. Semithin (0.5 μm) and ultrathin (80 nm) sections were cut with a Reichert-Jung Ultracut E ultra-microtome (Leica Microsystems GmbH, Germany). Toluidine blue (1%) stained semithin sections were examined to select the appropriate location for ultrathin sections. The latter were mounted on copper grids, contrasted with lead citrate and uranyless (EMS, USA), and examined by TEM (Philipps CM12).

## Results

### Macroscopical findings

**Figure 2:**
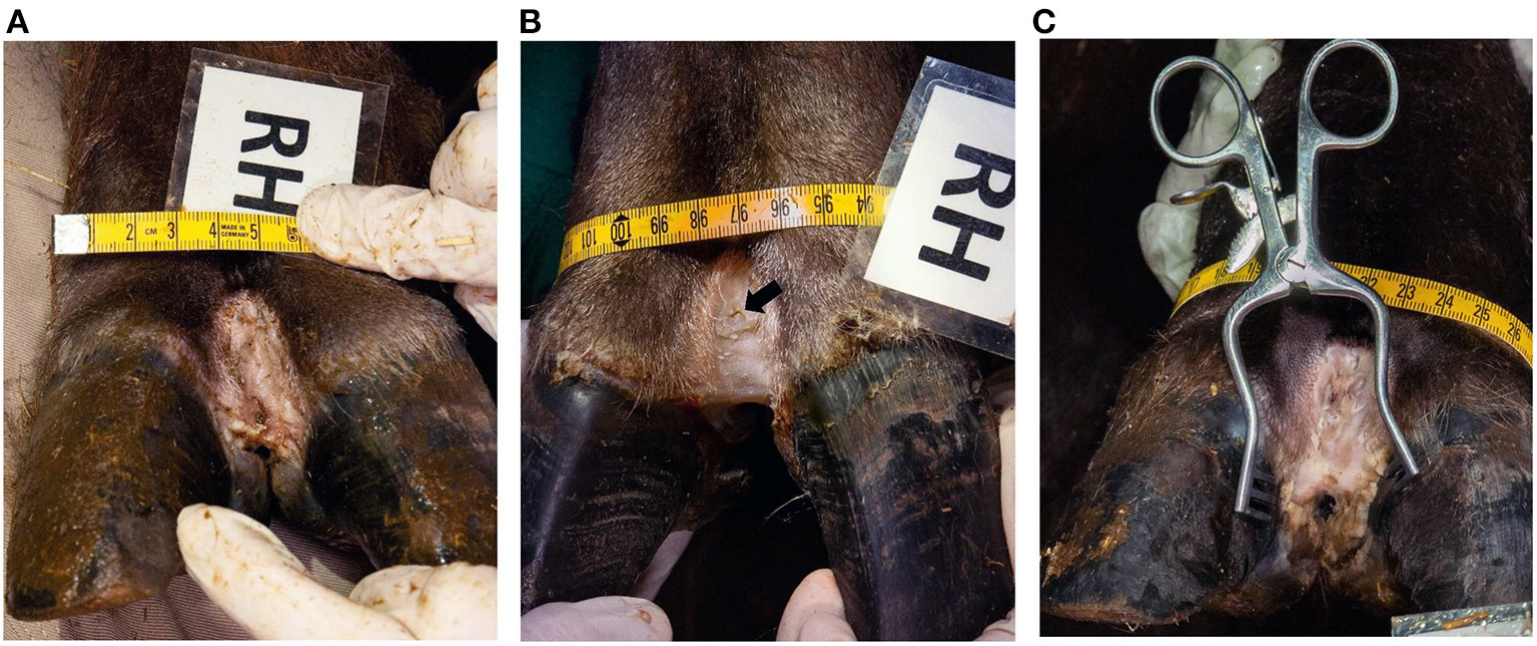
Clinical appearance of dorsal interdigital cleft of the right hind foot (RH) of animal No. 4. **(A)** Before treatment (macroscopical score 2/2), **(B)** after treatment (macroscopical score 0/0) (the arrow shows the biopsy site B1, taken before treatment), **(C)** and 2 months after treatment (macroscopical score 1/2).

In contrast to the brief clinical assessment, where all feet were assessed as DD affected pre-treatment, 15/20 were scored with lesions in the subsequent detailed scoring based on the photos. After treatment, the 15 feet were clinically healed (score 0/0), leading to a recovery rate on foot level of 100%, with a 95% confidence interval of 80–100%. The left hind foot of animal No. 5 was initially scored 0/0 but presented a thin layer of hyperkeratotic tissue (score 1/2) after treatment. An overview of the results is given in Table 1. The macroscopical lesions ranged from hyperkeratotic pale to slightly reddened skin and smeary plaques. They were observed focally on the dorsal aspect of the digital skin, in some cases extending into the interdigital cleft (Figure 2A). After treatment, most of the hyperkeratotic tissue could be manually removed, leaving a new epithelial layer with a smooth skin appearance, partly still with small protruding skin areas that could not be removed (Figure 2B).

Animals No. 2 and 5 were re-anesthetized five months, and animal No. 4 two months after treatment for transport reasons. Only a small lesion (score 1/1) could be detected in animal No. 2 on the right hind foot. Animal No. 5 showed small lesions (score 1/1) on the right front and left hind, and more extended lesions (score 1/2) in the right hind foot. The left front foot was still without lesions. In animal No. 4 the lesions reoccurred on all feet with a score of 1/2 in the right hind foot (Figure 2C) and 2/2 in the other three feet.

### Histological findings

A summary of the histological findings is provided in Table 1. All biopsies taken before treatment (B1) showed either a mild (*n* = 12) or moderate (*n* = 5) chronic perivascular and lymphoplasmacytic dermatitis except for three biopsies originating from animals No. 2 and 4, where no changes were present. After treatment (B2), dermatitis was still present in 12/20 specimens. Except for animal No. 2 in which the two right feet revealed higher scores for dermatitis after treatment, all other 18 feet were found to have the same (*n* = 9) or a lower (*n* = 9) degree of dermatitis after treatment. Keratinolysis characterized by intracellular edema, degeneration, and lysis of the corneocytes was only present in three animals before treatment. In the WS stain, spirochetes were found as black, slender, elongated rods in animal No. 3 (left hind), 4 (right hind), and 5 (right front and hind). In animal No. 3 and 4, spirochetes were invading the epidermis down to the stratum granulosum in one foot each, whereas in animal No. 5, spirochetes were found on top of the stratum corneum in two feet. After treatment, spirochetes were not found in any of the samples. Erosion or ulceration could not be detected in any of the specimens, neither before nor after the treatment.

### PCR and sequencing results

*Treponema* spp. DNA was detected by TT-PCR in 17/20 (85%) of the biopsies taken before and in none of the biopsies taken after treatment (Table 1), revealing a bacteriological healing rate of 100% on foot level with a 95% confidence interval of 82–100%. All positive TT-PCR products were further analyzed by sequencing, revealing identities > 97% in 15/17 samples to the following *Treponema* phylotypes: The *Treponema* phylotype PN-20 [GenBank accession number GQ424185 (34)], which was the most abundant (65% of all positive TT-PCR samples), and the *Treponema* phylotype PT3 [GenBank accession number AM942447 (28)], which could be found in four feet (24%). *D. nodosus, F. necorphorum*, and *P. levii* could not be detected by PCR in any of the samples.

### FISH results

The presence of *Treponema* spp. could be visualized by FISH in all hind feet (*n* = 10) and two front feet before treatment (Table 1). *Treponema* spp. were visible as spirochetal forms on top of the epidermis, and in the superficial part of the stratum corneum to a moderate or high number. After treatment, *Treponema* spp. could not be found in any of the examined biopsies, leading to healing rate of 100% at foot level with a 95% confidence interval of 76–100%. Occasionally, other bacteria such as cocci and rods were hybridized and could be found in some of the samples.

### TEM results

**Figure 3:**
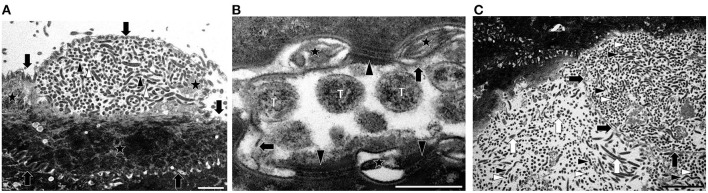
Transmission electron microscopy (TEM) images of spirochetes from animal No. 5 illustrating the morphology and localization in the infected tissue. **(A)** Transmission electron micrograph of spirochetes infecting a corneocyte on the surface of the stratum corneum. The membrane of the cell is marked with arrows. Note that the spirochetes (white arrowheads, transverse sections; black arrowheads, longitudinal sections) have not yet invaded the entire cell, and that keratin is still present (stars) (TEM. Magnification: 5.600x, the bar represents 2 μm) **(B)** Transmission electron micrograph showing cell membrane damage concurrent to spirochetal infection of a corneocyte. Transversely (T) sectioned spirochetes are visible. The intercellular space is dilated, contains electron-dense material (stars) and the cell membrane of the infected corneocyte shows discontinuities (arrows). Note that there are still intact corneodesmosomes (arrowheads) (TEM. Magnification: 53.000x, the bar represents 500 nm). **(C)** Transmission electron micrograph of spirochetes on the surface of the stratum corneum and inside a corneocyte. There are many spirochetes (white arrowheads, transverse sections; black arrowheads, longitudinal sections) on the surface and inside a largely infected corneocyte. The membrane of the conreocyte is marked with black arrows. On the surface of the stratum corneum smaller amounts of other unspecific bacteria (white arrows) are visible (TEM. Magnification: 3.400x, the bar represents 5 μm).

TEM revealed the presence of spirochetes in two feet of animal No. 5 (right hind and left front). The bacteria with the typical coiled protoplasmic cylinder and periplasmic flagella under the outer membrane were detected in the upper layers of the stratum corneum and found inside corneocytes (Figure 3A). While the cell membrane of the infected corneocytes was more or less intact, their cornified cytoskeleton was only rarely visible, giving infected cells a more electron-lucent appearance than non-infected neighboring cells or non-invaded regions of infected cells (Figure 3A). The inter-cellular space between corneodesmosomes was dilated and filled with electron-dense material; discontinuities in the membrane of infected corneocytes were occasionally noted (Figure 3B). The outer surface of the samples was covered with large numbers of free (non-cell associated) spirochetes and cellular debris, as well as additional bacteria in smaller numbers (Figure 3C). Post- treatment, no spirochetes could be detected by TEM.

## Discussion

This is the first study to evaluate treatment of DD in European bison. Promising results could be shown after a single treatment with SA under firm bandages over a 7-day period. Macroscopic healing occurred, and absence of pathogen DNA was proven by *Treponema* specific PCR and FISH in all tested five animals. The foot bandages were well-tolerated with no changes in gait or behavior. Therefore, this topical, non-antibiotic treatment can be recommended in that species.

The treatment protocol was adopted from a cattle study (24), where repetitive SA paste bandages with an interval of 7 days led to 100% healing rate of the 26 ulcerative lesions treated for 2–4 weeks. In our study, all 15 clinical lesions from five European bison healed after only 7 days of SA treatment. In this study, all feet were biopsied and treated with a povidone-iodine ointment bandage for 1 week as a precautionary measure to prevent infection post biopsy. It may be speculated that this experimental procedure (administration of povidone-iodine ointment bandages before SA treatment) had an impact on the healing process, as discussed earlier (24). An additional week of treatment may be required if no povidone-iodine bandage is applied beforehand. The efficacy of SA under bandages with a different interval of four and followed by 10 days was also tested to treat chronic DD lesions in cattle, but with lower success rates of 44% after 2 weeks (26).

Treatment was applied on all four feet of all animals to avoid overlooking subclinical infection, resulting in an unexpected outcome. The left hind foot of animal No. 5 was considered healthy before treatment, but a focal hyperkeratotic lesion was present after removal of the demarcated skin after treatment which was classified as DD lesion. As no *Treponema* spp. could be detected in that foot, we concluded that the post-treatment macroscopical findings might have been an iatrogenic skin irritation due to the keratolytic effect of SA instead of a DD lesion.

Most DD treatment studies verify their efficacy based on macroscopical evaluation of lesions and lameness scoring (35–37). Ideally, however, animals should be treated before lameness occurs, and before treponemes already infiltrate deeper layers of the skin, where topical medications are less effective (38). No abnormal gait or lameness could be linked to the lesions in European bison neither in this nor in the previous study (11). As pain evaluation through palpation cannot be assessed in an anesthetized animal treatment success was evaluated by pre- and post-treatment biopsies, based on a template of a former study (24).

Histologically, dermatitis was detected in 17/20 feet and keratinolysis in 3/20 feet before treatment. Whereas, 12/20 feet still showed dermatitis after treatment, keratinolysis was not detectable anymore. Similar findings were described in cattle, where 77% of the macroscopically healed lesions were still associated with dermatitis histologically, but 0% with keratinolysis (24). These findings indicate that wound healing is still ongoing, despite the macroscopical appearance of healed lesions and pathogen absence.

Sequencing of the positive PCR results revealed two *Treponema* phylotypes: *Treponema* phylotype PN-20 (GQ424185) and *Treponema* phylotype PT3 (AM942447). Both belong to the *Treponema refringens* phylogroup and were associated with DD lesions in cattle (13, 28, 34). Depending on lesion stage, the dominant phylotypes can vary in bovine DD, whereas *Treponema* phylotype PT3 has been found belonging to the phylotypes dominating the early lesion stages (39). This agrees with the generally mild clinical lesions detected in the European bison of this study. By FISH, the hybridized *Treponema* spp. were exclusively visualized in the superficial layers of the stratum corneum to a moderate to high amount. In former studies, phylotypes of the *Treponema refringens* phylogroup appeared to be located more superficially compared to other phylogroups (28, 34), which supports our findings of only superficial tissue infiltration.

The ultrastructural examinations carried out in animal No. 5 confirmed the presence of treponemes on the surface and in the upper layers of the stratum corneum. Interestingly, the treponemes were intra-cellular, within cornified keratinocytes (corneocytes), and not in the intercellular space, as seen in previous TEM examinations of DD-affected cattle (40, 41). In addition, the electron-lucent appearance of the infected cells indicates that the treponemes disrupt the cytoskeleton.

No *Treponema* spp. could be detected after treatment by WS, PCR, FISH, and TEM. Nevertheless, three animals re-examined 2–5 months after the end of the study were found with recurrent clinical lesions in one to four feet. Because the animals were kept in an infected herd (11), reinfection was anticipated. The wound created by the post-treatment biopsy may have facilitated reinfection. Recurrence after SA treatment is also described in other studies, in which rechecks occurred within 7–17 days (26) and 15–22 weeks (24).

The three bacterial species *D. nodosus, F. necorphorum*, and *P. levii* are among others suspected to have a synergistic role in the onset and progression of DD lesions in cattle (39, 42). As they could not be detected in any of the five animals from our study, they are not likely to contribute to the DD in those European bison. Further studies on the etiopathogenesis, risk factors, and prevalence of DD in European bison are needed to (a) better understand the impact of these lesions in the newly discovered host species and (b) precisely define the indications for the use of personnel- and time-intensive treatment regimens. As a species that is regularly transferred between institutions and rewilded in Europe, preventing the transmission of infectious diseases is of particular importance (43). The rapid spread of *Treponema*-associated hoof disease in free-living elk illustrates the difficulty of controlling this infectious hoof disease once it has established in the population (44).

A limitation of the study is the low number (*n* = 5) and the lack of control animals included in this study, especially as bovine DD lesions have been reported to heal spontaneously (45). However, due to the risk and logistical effort associated with three general anesthesias at weekly intervals, it was not justifiable to include additional animals from the Berne Animal Park.

In conclusion, the first attempt to treat chronic DD lesions in anesthetized European bison with SA under bandages was successful. The results imply follow up studies to test this treatment protocol in other affected species. In this trial, the application of SA under bandage for 7 days revealed clinical healing without evidence of *Treponema* spp. by WS, PCR, FISH, and TEM. We recommend it as standard procedure for affected European bison before transportation to minimize the risk of disease spread, especially for animals that are released into the wild.

## Data availability statement

The original contributions presented in the study are included in the article/Supplementary material, further inquiries can be directed to the corresponding author.

## Ethics statement

The animal study was reviewed and approved by the Animal Experimentation Committee of the Canton of Bern (BE10/21, 33227).

## Author contributions

The conceptualization including the animal experiment application was done by SJ, MA, AS, BF, and SH. The animal experiments were carried out by SJ, MA, TZ, and SH. Laboratory processing and evaluation of PCR were performed by SJ, MA, and SB, FISH samples by SJ, MA, and TJ. Histological examinations were done by CG, and electron microscopical examinations by TZ and SK. BF analyzed the data statistically. SJ wrote the initial version of the manuscript. MA, AS, and SH edited the manuscript and supervised the study. All authors contributed to the article and approved the submitted version.

## Funding

This work was supported by the foundation of the Animal Hospital in Basel, the Eva Husi foundation, and the Stotzer-Kästli Foundation.

## Conflict of interest

The authors declare that the research was conducted in the absence of any commercial or financial relationships that could be construed as a potential conflict of interest.

## Publisher's note

All claims expressed in this article are solely those of the authors and do not necessarily represent those of their affiliated organizations, or those of the publisher, the editors and the reviewers. Any product that may be evaluated in this article, or claim that may be made by its manufacturer, is not guaranteed or endorsed by the publisher.
